# Assessment of Alternative Body Points for Temperature Screening As Precautionary Screening During the Pandemic Using Infrared Thermometry

**DOI:** 10.7759/cureus.31712

**Published:** 2022-11-20

**Authors:** Ananyan Sampath, Santosh Wakode, Ragini Shrivastava, Tanusha Pathak, Avinash Thakare, Naina S Wakode

**Affiliations:** 1 Medicine, All India Institute of Medical Sciences, Bhopal, IND; 2 Physiology, All India Institute of Medical Sciences, Bhopal, IND; 3 Anatomy, Atal Bihari Vajpayee Government Medical College, Vidisha, IND

**Keywords:** alternative body points, mass screening, temperature screening, body temperature, clinical thermometry, infrared thermometry

## Abstract

Background: The recent coronavirus disease 2019 (COVID-19) pandemic, which swept across the globe in a short period, demonstrated that disease transmission management is a critical step in preventing an outbreak, as is good viral infectious disease screening. Infrared thermography (IRT) has long been considered ideal for screening body temperatures during pandemics.

Methods: Single-centre cross-sectional study with 159 participants. Using infrared thermometry, participants were subjected to temperature measurement twice daily on various sites. This was compared to oral temperature.

Results: The findings of the study revealed that infrared thermometry could be utilised as a proxy approach for screening by both individuals and medical professionals when employed at the glabella, cubits, or axillae.

Conclusion: Temperature screening is implied as a prophylactic method during pandemics. Owing to contact limitations, oral thermometry cannot be used for mass screening during the pandemic. Infrared thermometry is a noncontact method of temperature screening that can readily be applied for mass temperature screening in congested venues such as airports, shopping malls, places of public convenience, and other similar locations.

## Introduction

The emergence of the SARS virus in 2003 pushed several nations to adopt border control measures. Thermal screening - via thermal scanners (infrared thermal imaging systems) and handheld, non-contact infrared thermometers (NCITs) - is deemed the safest tool for screening temperature during infectious disease outbreaks such as SARS,1 H1N12,3 and presently, COVID-19 [1]. The World Health Organization has implemented public health measures, such as body temperature screening to detect probable coronavirus cases quickly and prevent infection [2,3].

Since the start of the COVID-19 pandemic, thermal detection has been used to monitor body temperature as a primary prophylactic measure to keep the SARS-CoV-2 virus from spreading [4,5]. Temperature measurement devices range from traditional mercury-in-glass thermometers, electronic digital readout thermistors, infrared thermometers for the skin, tympanic cavities, zero-flux thermistors, and thermal imaging cameras. In a healthy person, the average body temperature for an adult is 98.6°F (37℃), but it can be as low as 96.8°F (36℃) or as high as 99.5°F (37.5℃). Upward deviation from this range indicates fever and may require treatment [2]. Although temperature screening is not an effective way to avert an epidemic due to its low specificity, it can likely be used as part of a primary screening because of its increased sensitivity. This could be due to inadequate instrumentation and calibration methods and a lack of acceptable, regularly executed standardised deployment and screening procedures [5,6].

Invasive and non-invasive approaches can be used to determine body temperature [7]. Blood temperature is the gold standard for establishing core body temperature, as reported by Shellock and Rubin and Fulbrook [8,9]. Other invasive procedures, such as the oesophageal, nasopharyngeal, or urinary bladder, monitor core body temperature at various places [10]. Rectal temperature measurement is considered the most reliable as the ambient air temperature has negligible influence, but it is the least practical among others [11].

Nearly all such instruments are accurate to 0.1℃. In routine clinical practice, oral (sublingual) or axillary (armpits) are preferred sites for body temperature measurement. Recent literature does show promising evidence of axillary temperature as a reliable predictor of core body temperature [12-15]. While these procedures accurately represent core body temperature, their intrusive nature limits their use in a broad spectrum of patients during outbreaks or pandemics like COVID-19. Infrared thermometry is the least invasive as it is non-contact and measures body temperature from < a 5-cm distance [2].

Since the COVID-19 pandemic has amended routine temperature screening practices, infrared thermometers have become the mainstay for temperature screening in all public areas to avoid contact surfaces and the risk of transmission. Infrared thermography (IRT) measures the emitted infrared radiation from the skin's surface [16,17].

Like most other biological variables, it is essential to note that body temperature fluctuates throughout the day, attributable to the circadian rhythm and cyclic hormone release [18]. However, the pattern of variation is consistent, with the lowest temperatures occurring between 3 and 6 a.m. and the highest occurring between 3 and 6 p.m. [19]. Physiological parameters such as age, gender, food, body mass, hormonal state, pathological states, and pathological conditions influence core body temperature in a human [19]. The current study will compare the body temperature measured by infrared thermography at multiple sites to the oral and axillary temperatures measured by reference contact clinical thermometers while accounting for the circadian rhythm and external environment.

## Materials and methods

This is an observational, analytical, prospective cross-sectional study conducted at a single tertiary centre in Central India. The study uses the purposive convenience sampling method. Hetaida Carent, Model HTD8813C, non-contact infrared thermometer and Hicks digital contact thermometer, used as a reference thermometer. The study was carried out in July, as local variations are least during this time, the room temperature was maintained in both situations (morning and evening), and 30 minutes of acclimatization time was given to each participant after entering the room before the study. The purposive Convenience Sampling method was used.

Participants

**Figure 1:**
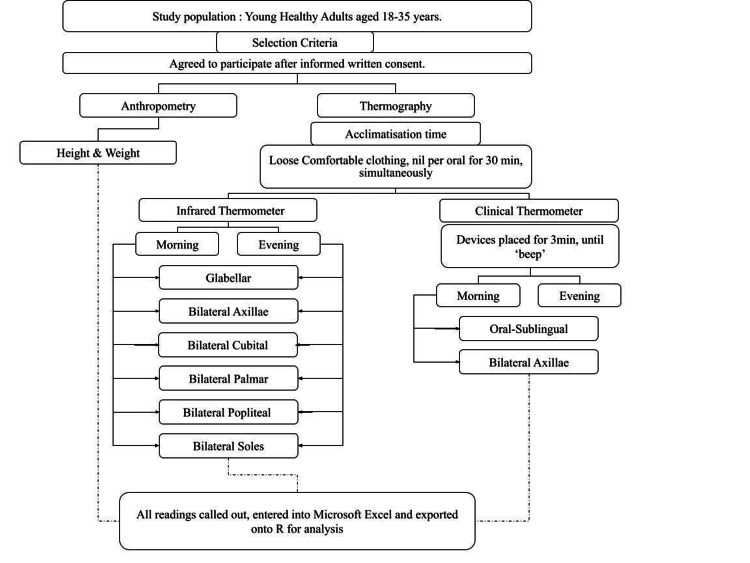
Study flow-chart

One hundred fifty-nine adults between the age group of 18-35 years were recruited for participation. Exclusion criteria included a history of smoking, alcohol intake and consumption of centrally acting drugs or antipyretics or oral contraceptive pills recently or were suffering from known illnesses that may cause temperature variation or were menstruating in the preovulatory phase of the menstrual cycle or were pregnant. Also, individuals with any obvious local inflammation or pain in the sites of temperature measure generalised elevation in body temperature secondary to infection or inflammation were excluded. Figure 1 illustrates a detailed study protocol.

Procedure

All participants, after informed written consent, were subjected to measurements of basic anthropometric measurements, where their height and weight were measured to the nearest centimetre and gram, respectively.

Thermography

Before temperature measurement/recording, participants were instructed to wear loose & comfortable clothing before coming to the temperature screening room and not to consume any centrally acting drugs or caffeine before 12 hours of temperature measurement. Participants were brought to a temperature-controlled room common to all and were given 30-minute acclimatization to avoid variations due to the ambient temperature. 

All thermometers were calibrated using ice water. Clinical or oral thermometers were disinfected using cold water and rubbing alcohol before each reading. Temperature measurements were recorded simultaneously to the nearest 0.1℃, in duplicates and later averaged out within three minutes (each set) via the infrared thermometer and contact thermometer to avoid environmental and subject variations twice during the same day between 06:00-08:00 hours and 18:00-20:00 hours to account for the circadian rhythm's effects on body temperature.

Infrared thermography

Temperatures were recorded using the infrared thermometer from the glabellar region, bilateral axilla, bilateral cubital fossa, a bilateral centre of palms, bilateral popliteal fossa, and bilateral soles as per the operation guidelines specified for the device, 2.5 cm from the surface of the skin. Readings were called out and immediately recorded and manually entered into an excel sheet.

Clinical thermography

**Figure 2:**
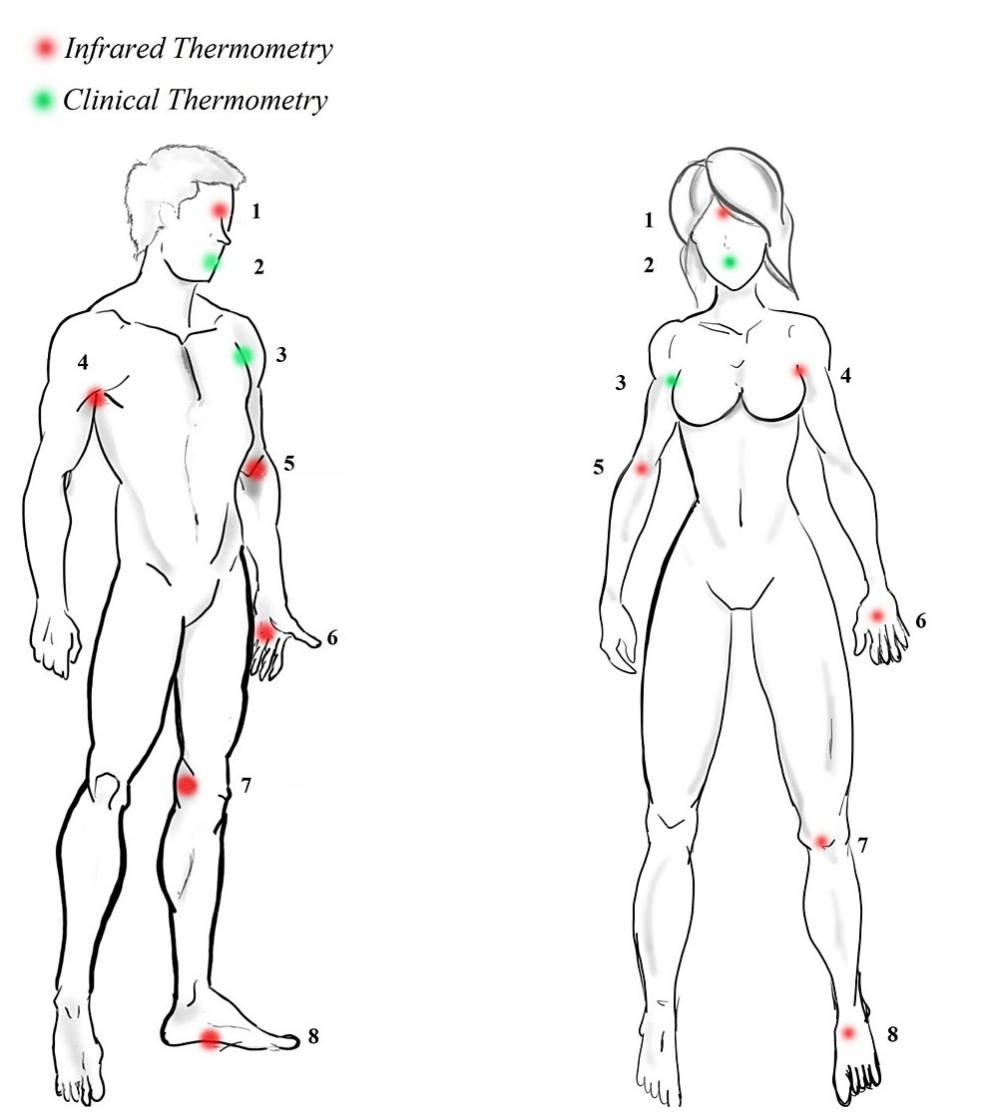
Alternative points for temperature screening 1. Glabella                                                                   5.   Bilateral Cubital 2. Oral-Sublingual                                                       6.   Bilateral Palmar 3. Bilateral Axillae                                                      7.   Bilateral Popliteal 4. Bilateral Axillae                                                      8.   Bilateral Soles

The three contact reference thermometers were used to record the sublingual temperature (with the lips pursed and the root of the tongue in contact, and the inferior surface of the tongue supporting the instrument) and bilateral axilla (with the long axis of the thermometer pointing towards the apex of the cervical-axillary canal in contact with the skin) simultaneously until the device “beeped” (Figure 2) [20].

Data analysis

Data were collected using paper-based tools and compiled on (Microsoft Excel) Computer-based spreadsheet. Subsequently, the data was cleaned and uploaded to R studio (version ver.3.1.1) using the packages gtsummary, ggplot2 and tidyverse for data analysis. For the parametric data, the central tendency of dispersion has been represented as Mean (SD); for non-parametric data, the central tendency has been expressed as median (IQR). Welch's two-sample t-test was used to compare differences in temperatures between different sides and at the same site at the same time. P-values < 0.05 were considered to be significant.

Ethical consideration

The study was conducted after obtaining ethical clearance from the Institutional Human Ethics Committee of AIIMS Bhopal (IHEC) AIIMS Bhopal vide permission number (IHEC) IHE-LOP/2020/IM0351.

Statistical analysis

All data analysis was done by R version 4.0.3 (R Core Team 2020). Temperature data reported by participants were entered into a Microsoft Excel spreadsheet and then checked for redundancies concerning implausible values. Temperature data were summarized using mean and standard deviation, and its distribution is presented as density plots or boxplots stratified by site and timings. Oral temperature measured by clinical thermometer was considered reference temperature for comparison of temperature measured at different sites by clinical and infrared thermometers. The correlation between oral temperature and peripheral temperature was tested using Pearson's correlation coefficient and the Concordance Correlation Coefficient. Linear regression analysis was performed for clinical thermometry, where the oral temperature was entered as a dependent variable while the axillary temperature was an independent variable. The difference in temperature of a given site for different measurement timings was calculated, and its distribution is shown using boxplots. Bland-Altman plots showing differences from the clinical oral temperature on Y-axis and reference temperature, i.e., clinical oral on X-axis, were also generated.

## Results

A total of 159 participants were enrolled in the study, of which 102 (64.2%) were females, and 57 (35.8%) were males. Table 1 shows the mean and standard deviation of temperature measured at different sites, times and methods.

**Table 1:** Distribution of temperatures by method, site, side, and time

Temperature recording method & Site	Morning		Evening	
	Left	Right	Left	Right
Clinical Oral	97.9 (1.0)		98.2 (0.9)	
Clinical Axilla	98.0 (0.8)	98.0 (0.9)	98.3 (0.9)	98.3 (0.9)
IR Glabellar	98.1 (0.6)		98.3 (0.5)	
IR Cubital	97.8 (0.7)	97.9 (0.7)	97.9 (0.6)	97.9 (1.0)
IR Popliteal	97.3 (1.5)	97.2 (1.6)	97.1 (2.0)	97.2 (2.0)
IR Palm	97.2 (1.9)	97.3 (1.6)	97.3 (2.3)	97.5 (1.2)
IR Sole	95.1 (3.6)	95.3 (3.3)	95.0 (3.9)	95.0 (4.0)
IR Axilla	98.2 (0.7)	98.2 (0.8)	98.7 (0.8)	98.7 (0.9)

The correlation of oral temperatures with other sites was evaluated using Pearson’s correlation coefficient and Lind’s concordance correlation coefficient. Table 2 shows diurnal variations in temperatures. Axillary temperature positively correlated with the oral temperature in both morning and evening and by both methods. Lind’s concordance correlation coefficient was also moderate for axillary temperatures. However, there was a poor correlation between other peripheral sites and oral temperature.

**Table 2:** Correlation coefficient, Lind’s concordance correlation coefficient for temperature recorded at oral and other sites

Temperature recording method & Site	Morning		Evening	
	r	ccc	r	ccc
Clinical Left Axilla	0.386	0.379	0.245	0.243
Clinical Right Axilla	0.434	0.428	0.228	0.226
Infrared Glabellar	0.026	0.021	0.136	0.11
Infrared Left Axilla	0.315	0.28	0.284	0.248
Infrared Left Cubital	-0.026	-0.025	0.124	0.106
Infrared Left Palm	0.06	0.045	0.079	0.048
Infrared Left Popliteal	0.135	0.112	0.248	0.152
Infrared Left Sole	0.212	0.07	0.242	0.066
Infrared Right Axilla	0.424	0.381	0.227	0.201
Infrared Right Cubital	0.102	0.094	0.077	0.072
Infrared Right Palm	0.055	0.045	0.142	0.109
Infrared Right Popliteal	0.192	0.149	0.181	0.112
Infrared Right Sole	0.064	0.023	0.172	0.046

Correlation analysis of temperature among non-ailing individuals using a statistical method is driven by the fact that the variation is within a very narrow range. Clinically non-significant variation can be defined as a difference within 0.5-degree Fahrenheit or 1-degree Fahrenheit. Table 3 shows the mean and standard deviation difference from oral temperature and the percentage of observations within 0.5- and 1-degree Fahrenheit from oral temperature.

**Table 3:** Mean and standard deviation of difference from oral temperature, percentage of observations within 0.5-degree F and 1-degree F of oral temperature

Temperature recording method & Site	Morning				Evening			
	Difference		Percent within		Difference		Percent within	
Mean	SD	0.5	1	Mean	SD	0.5	1
Clinical Left Axilla	0.106	1.018	45.912	76.101	0.105	1.111	47.799	73.585
Clinical Right Axilla	0.114	0.991	50.943	74.214	0.079	1.123	50.314	74.843
Infrared Glabellar	0.245	1.119	53.459	77.358	0.037	0.986	55.346	84.277
Infrared Left Axilla	0.347	1.024	42.138	71.698	0.450	1.043	42.138	75.472
Infrared Left Cubital	-0.037	1.226	46.541	71.069	-0.342	1.062	42.767	72.327
Infrared Left Palm	-0.614	2.078	36.478	66.667	-0.986	2.368	26.415	55.975
Infrared Left Popliteal	-0.547	1.696	44.025	68.553	-1.098	1.955	38.365	61.006
Infrared Left Sole	-2.720	3.568	10.692	30.818	-3.260	3.739	13.836	27.044
Infrared Right Axilla	0.355	0.954	42.138	74.214	0.455	1.126	38.994	72.327
Infrared Right Cubital	0.019	1.124	48.428	75.472	-0.323	1.311	41.509	72.956
Infrared Right Palm	-0.549	1.856	30.818	67.296	-0.762	1.445	30.189	55.975
Infrared Right Popliteal	-0.711	1.710	36.478	66.038	-1.067	2.056	34.591	59.119
Infrared Right Sole	-2.526	3.356	13.208	27.044	-3.247	3.990	11.950	27.673

**Figure 3:**
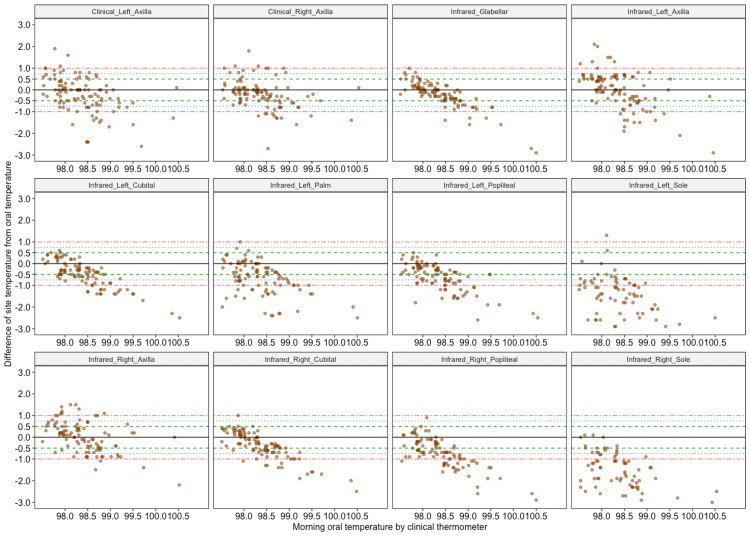
Bland-Altman plot showing clinical morning oral temperature and difference from it at different sites

**Figure 4:**
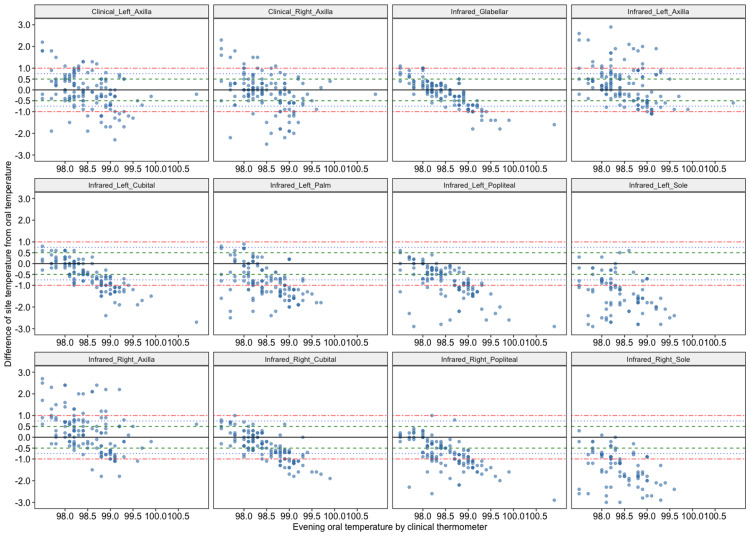
Bland-Altman plot showing clinical evening temperature and difference from it at different sites

Typical Bland-Altman plots are the scatter of the average of two measurements on the X-axis and the difference within them on the Y-axis. It is suggested to use reference measurement on X-axis. The standard deviation of the temperature difference for a site from oral temperature is approximately 1 degree Fahrenheit or more for most of the sites. Therefore, constructing a Bland-Altman plot where usual reference lines are +/- 2 standard deviations would show very good agreement for most of the sites; however, it implies a difference of 2 degrees which is not clinically acceptable. Thus, we have demonstrated reference lines at 0.5 and 1 degree to explore a clinically meaningful agreement. Figures 3, 4 show these for morning and evening, respectively.

**Figure 5:**
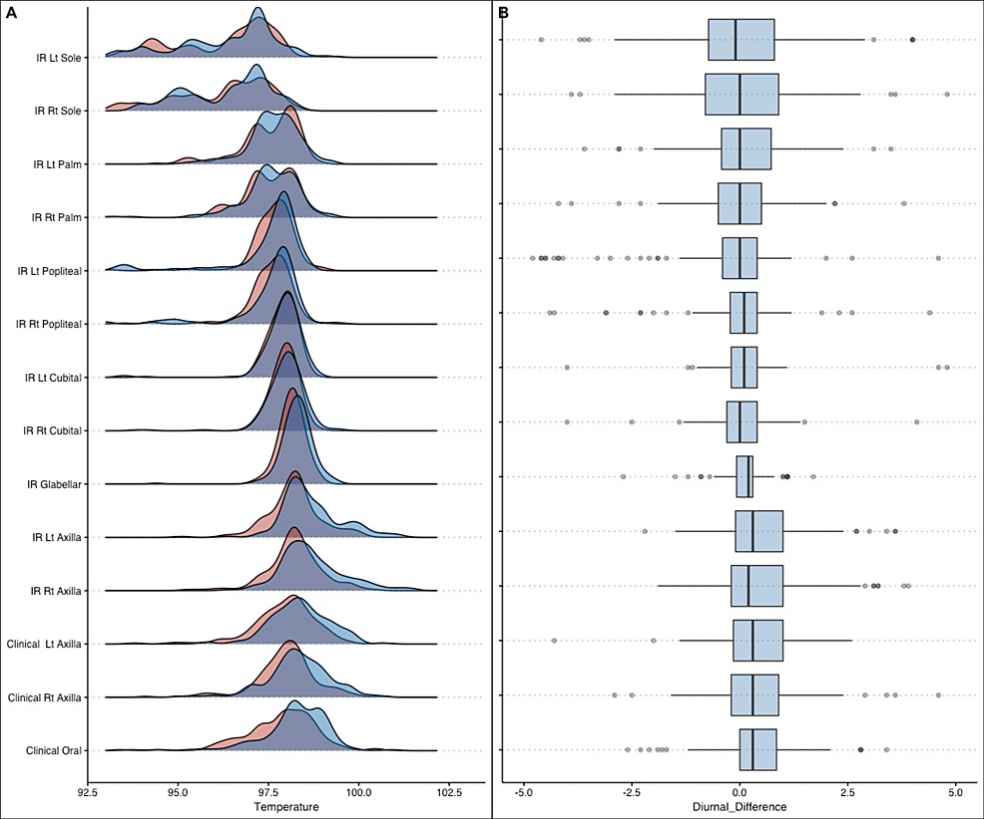
(A, B) Distribution of temperature at different sites by the time of measurement

From Table 3 and Figures 3, 4, it is evident that around 75% of participants had an axillary temperature (clinical thermometry) and glabellar temperature (IRT) within 1 degree Fahrenheit from oral temperature. These can be used when measuring oral temperature is not feasible. For the rest of the sites, variation was higher. These Bland Altman plots indicate the superiority of glabellar measurement as it shows lesser scatter around its mean. Figures 5A, 5B show the temperature distribution at different sites by measurement time. Mean evening temperatures were slightly higher at all sites.

The comparison between the morning and evening values of temperature for both Infrared and contact reference thermometer was plotted graphically to represent the sample distribution of each site and is described as an overlapping sample distribution curve, a general higher temperature (indicated by the right shift of the blue curves) of the evening temperatures as compared to the morning counterparts with most sites following a standard Gaussian distribution. The values of the soles and palms bilaterally show a wide distribution of values compared to the other sites, indicating a wider inter-individual variation. Temperature is expected to be similar at symmetrical sites.

Method comparison was also performed between the measurement techniques as follows: Difference in temperature among clinical and infra-red measurements at the same site and at the same time was also made. Table 4 depicts the difference and its confidence interval. IRT readings were consistently higher. However, the magnitude was smaller and clinically non-significant.

**Table 4:** Difference and confidence interval of temperatures among clinical and infrared measurements at the same site and the same time 1 - Mean (SD), 2 - Welch Two Sample t-test, 3 - CI = Confidence Interval

Characteristic	Infrared^1^	Clinical^1^	Difference^2^	95% CI^3^
Clinical Rt Axilla (M)	98.2 (0.8)	98.0 (0.9)	0.24	0.06, 0.42
Clinical Rt Axilla (E)	98.7 (0.9)	98.3 (0.9)	0.38	0.18, 0.57
Clinical Lt Axilla (M)	98.2 (0.7)	98.0 (0.8)	0.24	0.06, 0.42
Clinical Lt Axilla (E)	98.7 (0.8)	98.3 (0.9)	0.34	0.16, 0.53

**Figure 6:**
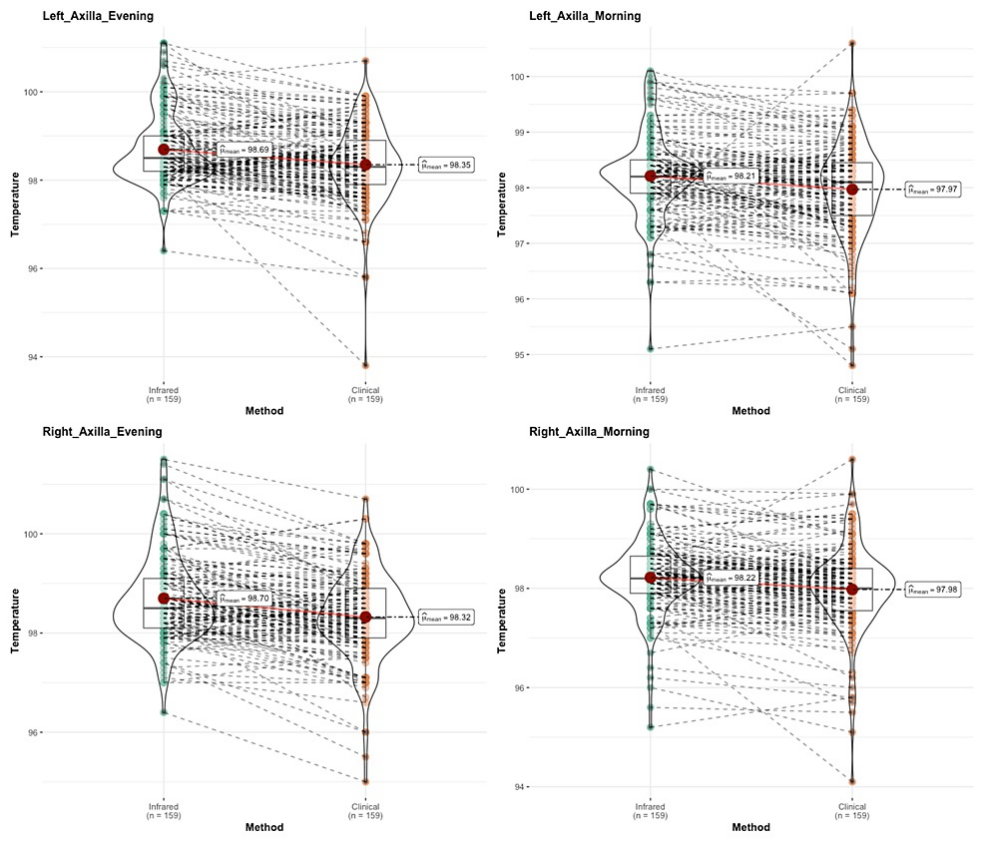
Individual diurnal axillary temperatures by IRT and clinical thermometry

Figure 6 depicts individual temperatures by IRT and clinical thermometry. Box-Violin plots show the distribution of temperature and summary measures. A dotted line connects each observation. Both methods have shown reliable results for axillary temperature, and a clinically non-significant difference was observed.

## Discussion

The global outbreak episodes of COVID-19 have demanded the use of quick and non-invasive methods of temperature screening, particularly at airports and border crossing locations. One of the ways described relies on remote-sensing IRT, a technique that has already been utilized to detect thermal anomalies linked to various inflammatory diseases [21].

However, there is very little research on the IRTs' accuracy and sensitivity on various detection sites compared to widely used contact clinical thermometers. The direct result was that axillary temperature, whether measured using infrared thermometers or contact clinical thermometers, both showed a parallel to the oral temperature and thus can be used as a viable site with similar accuracy. Yet, our study acknowledges that axillary temperature measurements are not always feasible in surveillance settings and necessitate a reliable temperature measurement method. One such suggested point is the glabellar temperature, which showed the slightest variation and is also a convenient method with much higher participant compliance, especially in regions of ethnic and cultural ideologies that prevent skin exposure other than the eyes for vision. Though our study did not find a significant correlation between oral-sublingual and glabellar temperatures, further studies with larger sample sizes and more complex study designs need to be conducted to ascertain its true potential, if any. Apa et al. and Sollai et al. assessed the clinical accuracy of non-contact infrared thermometers in paediatric settings and reported that IRTs are helpful in body temperature assessment [22,23].

In a recent study by Kameda, it was reported that temperature measured using a non-contact forehead infrared thermometer displayed a good correlation with the core temperature, and the correlation coefficient was higher than those for the temperatures measured using infrared tympanic and axillary thermometers [24]. The results revealed excellent accuracy comparable to tympanic temperature, with clinically sufficient precision. The accuracy and precision of infrared tympanic thermometers have also been previously reported by Kiya et al. and Masamune et al., and the results were in agreement with the results we noted in our study [25,26].

Another study by Chan et al. conducted a survey for passenger screening at airports using distant infrared thermography and found consistent correlations between readings from the side of the face with the core body temperature [21]. Multiple studies with a similar plan, i.e., mass screening for fever, were conducted during the SARS outbreak all arrived at similar conclusions about the usefulness of IRT as a proxy for the measurement of core body temperature [27,28].

However, a study by Liu et al. suggests that in outdoor situations, assessing the temperature of the auditory meatus is a more reliable screen for fever than detecting the forehead body surface temperature with the same infrared body thermometer [29]. This could be related to more prominent environmental factors affecting the forehead and body ambient temperature. Another study by Hausfater et al. also questioned the reliability of infrared thermometers, pointing out their low sensitivity [30].

The study being a cross-sectional single-centric study with a convenient sampling method that resulted in an uneven distribution of sexes in the sample population, selection bias may have affected the results. This study needs to be followed up by Cohort studies with larger populations and temperature control methods to validate the results. Furthermore, this study was conducted during a single weather period; thus, the varying thermoregulatory changes across climate and weather changes have not been accounted for.

## Conclusions

During pandemics, temperature screening is recommended as a possible means to prevent disease spread. Oral thermometry, despite being a standard method for determining the temperature in clinical settings, is not suitable for mass screening because of the constraints associated with its contact capabilities. Infrared thermometry is a non-contact method of temperature screening that is highly adaptable for mass temperature screening in crowded venues, such as airports, shopping malls, places of public convenience, and other similar locations. This study determined that infrared thermometry could serve as a proxy approach for screening by individuals and medical professionals when applied to the glabella, cubits, and axillae. In high-traffic areas, such as airports, train stations, hospitals, shopping malls, and other public transit hubs, these methods, which avoid physical touch, may function as safe screening procedures.
